# Cytocompatible liquid crystal elastomer fibers for potential application as artificial skeletal or cardiac muscle

**DOI:** 10.1038/s41598-026-54707-6

**Published:** 2026-06-04

**Authors:** Lukas Benecke, Dilbar Aibibu, Chokri Cherif

**Affiliations:** Institute of Textile Machinery and High Performance Material Technology, TU Dresden, Dresden, Germany

**Keywords:** Liquid crystal elastomer fiber, Artificial muscle, Biomaterial, Soft actuator, Biotechnology, Engineering, Materials science

## Abstract

To biomimetically mimic native muscle tissue new artificial soft actor materials need to be developed that can be processed into fibers and combine high mass-specific work, a sufficiently high contraction capacity, short contraction times, and biocompatibility. In this context, liquid crystal elastomers (LCE) are a promising class of materials that exhibit high actuator performance. Based on a novel spinning method to create MBB-based LCE fibers (LCEF), here we present an artificial muscle fiber capable of performing mass specific work of up to 31.2 J kg^−1^ while activation time was as fast as 0.466 s, resulting in an average mass specific power of 45.88 ± 23.75 W kg^−1^, thus to this extend mimicking skeletal muscle characteristics. Cell cytotoxicity assays were performed and compared to RM82-based LCEF from literature, showing that for the first time a cytocompatible LCEF was generated. Thus, the here presented actuator fibers offer great potential for future application in tissue engineering, e.g., as artificial skeletal or cardiac muscle.

## Introduction

Liquid crystal elastomers (LCE) combine the anisotropic orientability of liquid crystals (LC) in the liquid or dissolved state with the reversible deformability of weakly cross-linked polymer networks, the elastomers^1^. This unique combination enables the realization of outstanding property profiles, which are characterized by high, anisotropic mechanical properties as well as a material-intrinsic contraction capacity. Consequently, LCEs are promising materials for applications in e.g., soft robotics, sensor technology, architecture, medical technology and artificial muscles^2–5^.

Engineering science has always pursued the goal of observing, understanding and abstracting nature in order to solve technical problems using natural principles (bionics). In this context, the transformation of LCE into fibrous structures represents a necessary step towards developing actuators analogous to human muscles. Naciri et al. succeeded in producing liquid crystal elastomer fibers (LCEF) for the first time in 2003^6^. The fibers were produced by dipping metal tweezers into a partially pre-crosslinked LCE melt and pulling them out manually “as quickly as possible”. The short fiber fragments produced in this way exhibited pronounced irregularities. Since then, the development of LCEF has been driven forward with increasing interest.

LCEF fabrication methods described in the literature can be divided into three main categories: templates, 4D printing, and dry spinning. The use of templates or molds for shaping LCE is primarily known from the production of films. Various research groups have successfully applied this principle to the production of fiber fragments^7–12^. In most cases, tubular templates made of PTFE^12^ or glass^7,8,11^ are used. Furthermore, threaded rods^10^ and silicone molds^9^ have been successfully employed. The advantages of the template method lie in the simple experimental setups and the flexibility in adjusting the desired fiber geometry^13^. However, the method is discontinuous, and only short fiber segments can be generated. Furthermore, demolding poses a major challenge, as particularly surface defects often occur and lead to uncontrollable material behavior or failure. 4D printing refers to the additive manufacturing of three-dimensional objects with an intrinsic ability to change shape (4th dimension), which is programmed during the manufacturing process. In the case of LCE, only extrusion-based printing methods are used, such as Direct Ink Writing (DIW) or Electro Writing (EW)^14–17^. By precisely controlling shear forces during the printing process, areas with high and low contraction capabilities (or molecular orientations) can be flexibly combined into virtually any 3D pattern. In principle, continuous fiber deposition could be achieved e.g., via DIW onto a rotating collector roll^18^. However, the advantages of 4D printing are almost entirely lost in this process.

Conventional dry spinning is a subcategory of solvent spinning and is characterized by the solidification of the fiber after it exits the spinneret in a (usually) air duct through solvent evaporation. In the case of LCEF spinning, the solidification occurs through thermally^19,20^ or UV-induced^21–23^ cross-linking steps. Typically, the fibers generated in this process are first wound onto a (possibly heated) spool before being drawn out using additional spools. This involves a second (usually UV) cross-linking step to fix the nematic state of the LCEF. The alignment of the LC in the fiber direction is achieved, on the one hand, by shear forces in the nozzle^21^ and, on the other hand, during the stretching process^19–23^. The dry spinning processes described in the literature are characterized by continuous production of LCEF and the possibility of integrating fiber functionalization into the process. The integration of an electrically or thermally conductive network into the active fibers is often sought to enable active activation either directly through heating of the fibers or indirectly through Joule heating generated by electrical current. Hou et al., for example, integrated graphene into the spinning solution to create a percolation network^21^. Sun et al. generated sandwich structures consisting of two LCEFs with liquid metals enclosed between them through coating and lamination processes followed by stretching and UV cross-linking^23^. For this purpose, plasma treatment of the LCEFs was also performed to enhance surface adhesion. Similarly, Wu et al. functionalized the surfaces of their actuator fibers with electrically conductive MXenes using process-integrated plasma and coating treatments^19^. Electrospinning (ES) can also be considered a dry spinning process in a broader sense, since here too the fibers solidify in an air shaft due to solvent evaporation. What makes this process unique is the use of an electric field to generate the fiber jet. The extruded spinning solution is subjected to high voltage, causing charge carriers to agglomerate on the surface^24^. These repel one another. As soon as the electrostatic repulsive forces exceed the surface tension of the spinning solution, a jet is formed, which is accelerated toward a collector that is usually grounded or negatively polarized. In the process, the charges still immobilized on the surface repel each other, causing the fiber jet to thin out further. In addition, bending instabilities occur, which on the one hand lead to a randomized fiber arrangement in the form of a nonwoven fabric and on the other hand can reduce the resulting fiber diameters to as small as single-digit nanometers. He et al. used ES to produce LCEF nonwovens by combining the process with UV irradiation to cross-link and thus solidify the material^25^. The nonwovens were subsequently fractionated and stretched, and their ability to contract was successfully demonstrated. Wu et al. also succeeded in establishing an ES process capable of continuously producing LCEF microfiber bundles^26^. For this purpose, a rotating funnel-shaped collector was spun on both sides with LCE solution. As soon as a fiber deposited on the edge, it was twisted with additional fibers from the other spinneret due to the collector’s rotation. This twisted yarn was then wound up and UV-stabilized in the process. In a further step, the twisted yarn was drawn and finally UV-crosslinked.

So far, in the presented works mainly two liquid crystals or combinations of them are being used: 1,4-bis-[4-(6-acryloyloxyhexyloxy)benzeneoxy]-2-methylbenzene (RM82) and 1,4-bis-[4-(3-acryloyloxypropyloxy)benzeneoxy]-2-methylbenzene (RM257), which are also very similar in their chemical structure^7–10,12,14–17,19–23,25–27^. Although these works show promising actuation properties of the presented main-chain LCEF and outline their use as artificial muscles in biomedical applications, e.g. cardiac assistive devices, none of these works investigated cytocompatibility of these materials. In addition, there is still a significant need for research, particularly with regard to increasing the contraction frequency and mass specific power, in order to enable artificial muscles with a functionality comparable to human skeletal or cardiac muscles.

Recently, we presented a novel spinning method for preparation of 4-methoxyphenyl-4-(3-butenoxy)benzoate (MBB) fibers^28^, enabling for the first time the generation of a MBB-based LCEF. In contrast to RM82-/RM257-based LCEF spinning, this technology is able to process low viscosity spinning solutions through a multi-step crosslinking regime, highly enhancing material variability in future developments. Nevertheless, the generated side-chain MBB-LCEF did neither achieve actuation properties comparable to human muscle, nor RM82-/RM257-based LCEF from literature, thus general understanding of material-process-property-relations for MBB-based LCEF was needed.

Here, we present a new generation of MBB-LCEF that show similar mass specific power after actuation to human skeletal-muscle through variations of molar ratios of liquid crystal (LC) and elastomer in the spinning solution and advancements of the spinning technology. Furthermore, we evaluated cytocompatibility of the presented material and compared it to established RM82-based LCEF-compositions in accordance to DIN EN ISO 10993-5^29^. For the first time, a cytocompatible LCEF is presented that offers great potential for utilization in regenerative therapies, e.g., as artificial cardiac or skeletal muscle.

## Experimental section

### Materials for LCEF preparation

Liquid crystal 4-Methoxyphenyl 4-(3-butenyloxy)benzoate (MBB) and crosslinker 1,4-Bis(10-undecen-1-yloxy)benzol (11UB) were synthesized by GenoSynth GmbH (Berlin, Germany). Polydimethylsiloxane (PDMS), Polymethylhydrosiloxane (PMHS), Dichloro(1,5-cyclooctadien)platinum(II) (catalyst), 1,3,5-triallyl-1,3,5-triazine-2,4,6(1 H,3 H,5 H)-trione (TATATO), ethylenglycol-bis-mercaptoacetate (EGBMA), 2,2-Dimethoxy-2-phenylacetophenone (I-651), and toluene (≥ 99.5%) were purchased from Merck KGaA (Darmstadt, Germany). Liquid crystal 1,4-Bis-[4-(6-acryloyloxyhexyloxy)benzoyloxy]-2- methylbenzene (RM82) was purchased from Henan Daken Chemical Co., Ltd. (Hongkong, China). Silicone oil (PMHS, 350 cSt) was purchased from VWR International, LLC (Darmstadt, Germany). All materials were used as delivered without further purification.

### Preparation of MBB spinning solution

To prepare MBB spinning solution, MBB (870 mg, 2.91 mmol), 11UB (105 mg, 0.255 mmol), and PDMS (381 µl, 0.015 mmol) were mixed in 1.5 ml of toluene under constant stirring, thus generating a molar ratio of LC to crosslinker of approx. 1:0.09. After homogenization, 0.6 ml (10.21 mmol, 1:3.5) or 0.3 ml (5.10 mmol, 1:1.75) of PMHS and 1.845 ml of catalyst solution (25 mg of Dichloro(1,5-cyclooctadien)platinum(II) solved in 20 ml of toluene) were added to the solution. Afterwards, ultrasonic treatment was applied for 10 min. Finally, for degassing, the solution was placed in a desiccator and connected to a vacuum pump (VACUSAFE comfort, INTEGRA Biosciences GmbH, Biebertal, Germany), applying 0.3 bar for 10 min.

### LCEF spinning

Fiber spinning was performed as described in^28^. Briefly, a syringe pump (IPS14, Inovenso Inc., Cambridge, MA, USA) extruded the MBB spinning solution into a heated silicone oil bath (~ 90 °C), where first thermally induced crosslinking was achieved. Fibers were taken up at a speed of 1.15 m min^−1^ and gradually drawn by a factor of 2.3. During this process, the fiber was held above the heated oil bath, thus applying passive heating to achieve 2nd crosslinking. The as spun fibers of case group LCEF 1:1.75 received an additional drawing step post-spinning to enhance molecular orientation. Therefore, LCEF were stretched by a factor of 1.25 and held under tension for 24 h at room temperature until all solvent evaporated out of the elastomeric network. LCEF 1:3.5 could not be drawn post-spinning without fiber breakage.

### Preparation of RM82-based LCE for cell culture

Synthesis of RM82-based LCE was in accordance to^22^. At first, 3 g of the mesogen RM82 were melted in a 50 ml beaker in an oven (9010 − 0305, BINDER GmbH, Tuttlingen, Germany) at 110 °C. Afterwards, the beaker was placed on a stirring plate (444–0593, VWR International, Radnor PA, USA) preheated to 110 °C and 1.313 g of EGBMA (chain extender) as well as 0.278 g of TATATO (crosslinker) were added. After homogenization, 0.092 g of I-651 (2 wt% photo-initiator) were added and thin foils were cast and crosslinked through activation with UV-light (UV LED-strip 365 nm Protected, Luxalight B.V., Eindhoven, Netherlands).

### Characterization of mechanical properties

For characterizing mechanical properties, elastic moduli, tensile strengths, and elongations at break of the generated LCEF (*n* = 5) were determined by uniaxial tensile tests (ZwickRoell GmbH & Co. KG, Ulm, Germany) at a speed of 2 mm min^−1^, using a preload force of 0,05 N, 100 N load cell, and barometric clamps (1.5 bar). Per case group 8 specimen of length 100 mm were analyzed at room temperature and above T_NI_ at 60 °C (according to DSC measurements performed in^28^. Fiber diameter were determined prior to tensile tests for moduli calculation through image analyses of electron microscopy images (SEM, Quanta 250 FEG ESEM, FEI Company, USA).

### Characterization of contractile capacity and specific work

Contractile capability and mass specific work of LCEF performed during nematic-isotropic transition were determined in accordance to^28^. All LCEF specimen (*n* = 5) of approx. length 120 mm were weighed separately. Prior to testing, to eliminate irregularities resulting from defects in the spinning process, a preload of 25 mN was applied. Any samples that did not withstand this preload were excluded from the analysis. Afterwards, LCEF were placed into a heating chamber and nematic-isotropic transition was induced at 100 °C (at T_NI_ offset according to DSC measurements performed in^28^, to assure full contraction). Before and after applying temperature, fiber length was measured via image analysis using ImageJ. This procedure was repeated with increasing weights attached to the LCEF until fiber breakage occurred. Therefore, custom-made contacts were designed to safely attach additional weights starting at 1 g, assuring no fiber slippage during the process. Specific work was calculated, using Eq. 1:

1 $$w=\frac{{{m_w}*a* \Delta s}}{{{m_{LCEF}}}}$$

Here, w is the mass specific work of the LCEF performed during nematic-isotropic transition, m_*W*_ is the mass of the attached weight (including contacts), a is the gravitational acceleration (9.81 m s^−^²), Δs is the length difference of LCEF after nematic-isotropic transition, and m_*LCEF*_ is the mass of the LCEF.

### Determination of network density

Swelling tests were conducted to quantify the polymer network density and determine the average number of monomers between two network nodes. Foil specimen (*n* = 5) from all case groups were immersed in toluene for 20 h, and the increase in mass over this period was measured. Since all sample groups contain an identical molecular amount of 11UB crosslinking reagent, it is assumed that all the films produced have a comparable number of crosslinks. These crosslinks can link elastomer to LC as well as elastomer to elastomer, although only the latter contributes to an increase in network density and thus stiffness. Increased stiffness can hinder molecular orientation during drawing and is thus relevant for evaluating actuator behavior. Differences in swelling behavior can be attributed primarily to the average distance between these crosslinking points, which can be expressed as the average molecular weight between crosslinks (M_C_). This parameter was estimated using the Flory–Rehner equation (Eq. 2)^30,31^.

2 $${M_C}=\frac{{{\rho _{lce}}{V_s}\left( {{\phi ^{1/3}} - \frac{{2\phi }}{f}} \right)}}{{\ln (1 - \phi )+\phi +\chi {\phi ^2}}}$$

Here, ρ_lce_ corresponds to the density of LCE, V_s_ to the molar volume of the solvent (toluene), and f to the functionality of the crosslinking agent (two vinyl-groups). χ is a dimensionless parameter for polymer-solvent interaction according to Flory-Huggins and can be determined using Eq. 3^30,32^.

3 $$\chi =\frac{{{V_{lce}}{{({\delta _{lce}} - {\delta _s})}^2}}}{{RT}}$$

This parameter includes the molar volume of the LCE used (V_lce_) as well as the Hildebrand solution parameters of the LCE and the solvent, δ_lce_ and δ_s_, respectively. The gas constant (R) and the temperature (T) are also taken into account. The Hildebrand solution parameter is defined by Eq. 4.

4 $$\delta =\sqrt {\frac{{\Delta {H_v} - RT}}{{{V_m}}}}$$

In addition to the gas constant and temperature, the enthalpy of vaporization (ΔH_v_) and the molar volume (V_m_) of the respective reactants are taken into account.

ϕ (relevant for determination of M_C_ in Eq. 2) is defined as the reciprocal of the degree of swelling and can be determined using Eq. 5.

5 $$\phi =\frac{{{m_0}/{\rho _{lce}}}}{{{m_0}/{\rho _{lce}}+(m - {m_0})/{\rho _s}}}$$

Here, the densities of the LCE (ρ_lce_) and the solvent (ρ_s_) are compared to the initial weight (m_0_) and the change in weight after swelling (m–m_0_).

Since the LCE case groups under investigation are side-chain LCEs, and thus only the elastomer PMHS is relevant for the formation of the network, and since the solution parameters of the LC case groups under investigation were unknown, the calculation of M_C_ was performed using the density (1.006 g cm⁻³) and molar volume (58.78 cm³ mol⁻¹) of PMHS. The Hildebrand solution parameters are 9.588 cal^1/2^ cm^−3/2^ for toluene^33^ and 7.304 cal^1/2^ cm^−3/2^ for PMHS^34^. Using the molar mass of a PMHS monomer unit (59.13 g mol^−1^), the number of monomers between the network nodes was determined.

### Cytocompatibility study

To determine cytocompatibility, extracts of the LCEF had to be prepared. Beforehand, the as-spun fibers were immersed in ethanol for 24 h, which acts as a swelling agent and enables diffusion of unreacted reactants entrapped in the LCE network. For extract generation, 0.1 g LCE per case group (approx. three individual fibers of length 10 cm) were disinfected in ethanol again for 30 min and washed in 5 ml phosphate buffered saline (PBS). The samples were then incubated for 24 h at 37 °C in cell culture medium with fetal calf serum (RPMI 1640 MS01EB1001, BIOWEST SAS, Nuaillé, France) under constant agitation (rotary shaker Roto-Shake Genie, Scientific Industries Inc., Bohemia NY, USA). The extracts thus generated were then transferred to a cell lawn of fibroblasts (cell line L929) for 48 h. Afterwards, 2,3-bis-(2-methoxy-4-nitro-5-sul- fophenyl)-2 H-tetrazolium-5-carboxanilide (XTT reagent) was added to the samples. This chemical reacts with dehydrogenase enzymes to form a colored formazan, whereby the enzymes only occur in the metabolism of living cells. Colorimetric determination of the color change (Multiskan FC microtiter plate photometer, Thermo Fisher Scientific Inc., Waltham MA, USA) and comparison with data from extracts without cells and with cell lawns and XTT solution without extract allow statements to be made about cell viability as a result of LCEF contact.

Additionally, live-dead staining was performed using fluorescein diacetate (FDA) and propidium iodide (PI) (Merck KGaA, Darmstadt, Germany). Intracellular esterases of living cells hydrolyze FDA, enabling green fluorescence after excitation with light in the range of 450–490 nm. Dead and/or dying cells can be entered by PI, which binds to nucleic acids. Thus, after excitation at 530–585 nm dead cells fluorinate red. To prepare samples, cell culture medium was replaced by 100 µL of staining solution consisting of 0.8 µg of FDA and 2 µg of PI in serum-free RPMI 1640. Cells were incubated at room temperature for 5 min in the dark. After removal of the staining solution, cells were washed with PBS, and finally the medium without FCS was added to the samples. Fluorescence microscopy imaging (AxioVert.A1, Carl Zeiss Microscopy Deutschland GmbH, Oberkochen, Germany) of these prepared samples was used to evaluate cell phenotype.

### Contact angles

In addition to assessing cytocompatibility, the cell interaction of the fiber materials was further estimated by measuring their contact angle with deionized water using a tensiometer (K100. Krüss GmbH, Hamburg, Germany). Therefore, the Wilhelmy method for fibers as described in^35^ was applied. Briefly, five fiber fragments were attached to the immersion body in parallel configuration. Fibers were immersed in deionized water with a speed of 5 mm/min for a total depth of 5 mm. Contact angels were determined during immersion, whereas 50 individual measurements were conducted and averaged. For each case group three individual tests were conducted (*n* = 3).

### Nomenclature

This study presents three LCEF varying in the molar ratio of liquid crystal as well as post treatment (see section: Experimental). The case groups are named according to this molar ratio, e.g., if the molar fraction of the liquid crystal would be half of the elastomer, the case group would be referred to as “LC:elastomer 1:2”. A case group that exhibited a post-spinning drawing treatment receives the suffix “drawn”.

## Results and discussion

### Mechanical characterization

**Fig. 1:**
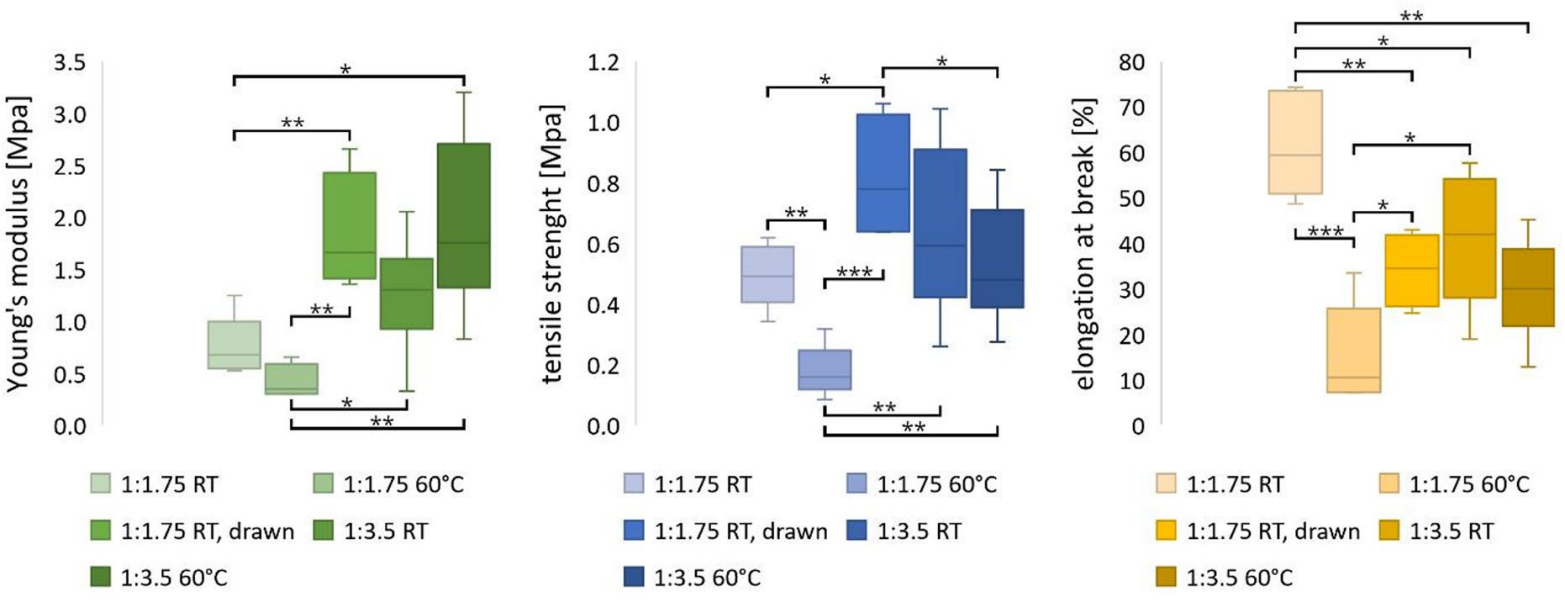
Mechanical properties of LCEF with varying elastomer content and draw ratios at temperature above (60 °C) and below (room temperature, RT) T_NI_; *n* = 5, statistical significance determined by a two-tailed t-test with confidence intervals of α = 0.05 [*], 0.01 [**], and 0.001 [***].

The mechanical properties have a considerable influence on the future applicability of the liquid crystal elastomer fibers (LCEF), as they are decisive for the load limits of the artificial muscles. At room temperature, the case group LC: elastomer 1:3.5 exhibited an average Young’s modulus of 1.26 ± 0.46 MPa and a tensile strength of 0.64 ± 0.24 MPa (Fig. 1). Above the nematic-isotropic transition temperature (T_NI_), an increase in Young’s modulus and a decrease in tensile strength to 1.94 ± 0.75 MPa and 0.52 ± 0.17 MPa were observed, respectively. The elongation at break decreased from 40.6 ± 13.2% to 29.4 ± 9.7% above T_NI_. The case group LC: elastomer 1:1.75 shows a similar behavior. After heating, the Young’s modulus increases from 0.75 ± 0.26 MPa to 0.84 ± 0.86 MPa. Tensile strength and elongation at break decrease above T_NI_ from 0.50 ± 0.09 MPa to 0.18 ± 0.08 MPa and from 61.62 ± 10.28% to 15.21 ± 9.92%, respectively. As a result of the post-drawing and storage of the freshly spun fibers under tension, the modulus of elasticity and tensile strength were also increased to values of 1.83 ± 0.49 MPa and 0.81 ± 0.18 MPa respectively. The elongation at break decreased to 34.12 ± 6.99% as a result of the post-treatment.

The contraction mechanism of the LCEF is based on a thermally induced molecular reorientation of the mesogen, as a result of which differences in the mechanical properties above and below T_NI_ are to be expected. Temperatures above T_NI_ lead to a loss of mesogen orientation and thus to an elongation of the elastomer backbone perpendicular to the orientation vector (longitudinal fiber axis)^36^. Since the molecular orientation is closely linked to the mechanical properties^37,38^, a decrease in tensile strength is to be expected after exceeding T_NI_, which was also demonstrated in this work. The change in the mechanical properties due to post-stretching and the resulting higher molecular orientation can also be explained. In contrast, the increase in Young’s modulus and the decrease in elongation at break were initially counterintuitive. The contraction of the LCE is entropy-driven^36^. During fiber production, the mesogens are oriented and this constrained state is fixed by the second crosslinking step. However, in order to maximize their entropy, the mesogens strive to adopt a lower order state, for which reorientation is necessary. To do this, they require thermal energy, which is achieved when the nematic-isotropic transition is crossed. During the tensile test, the LCEF, in particular their elastomeric backbone, are stretched, resulting in an alignment of the polymer chains that reduces the entropy of the overall system. Thus, in the tensile test above T_NI_, the reorientation of the mesogens exerts a counterforce to the fiber distortion, which can explain the increase in Young’s modulus and the decrease in elongation at break in LCEF after heating.

### Actuator properties

**Fig. 2:**
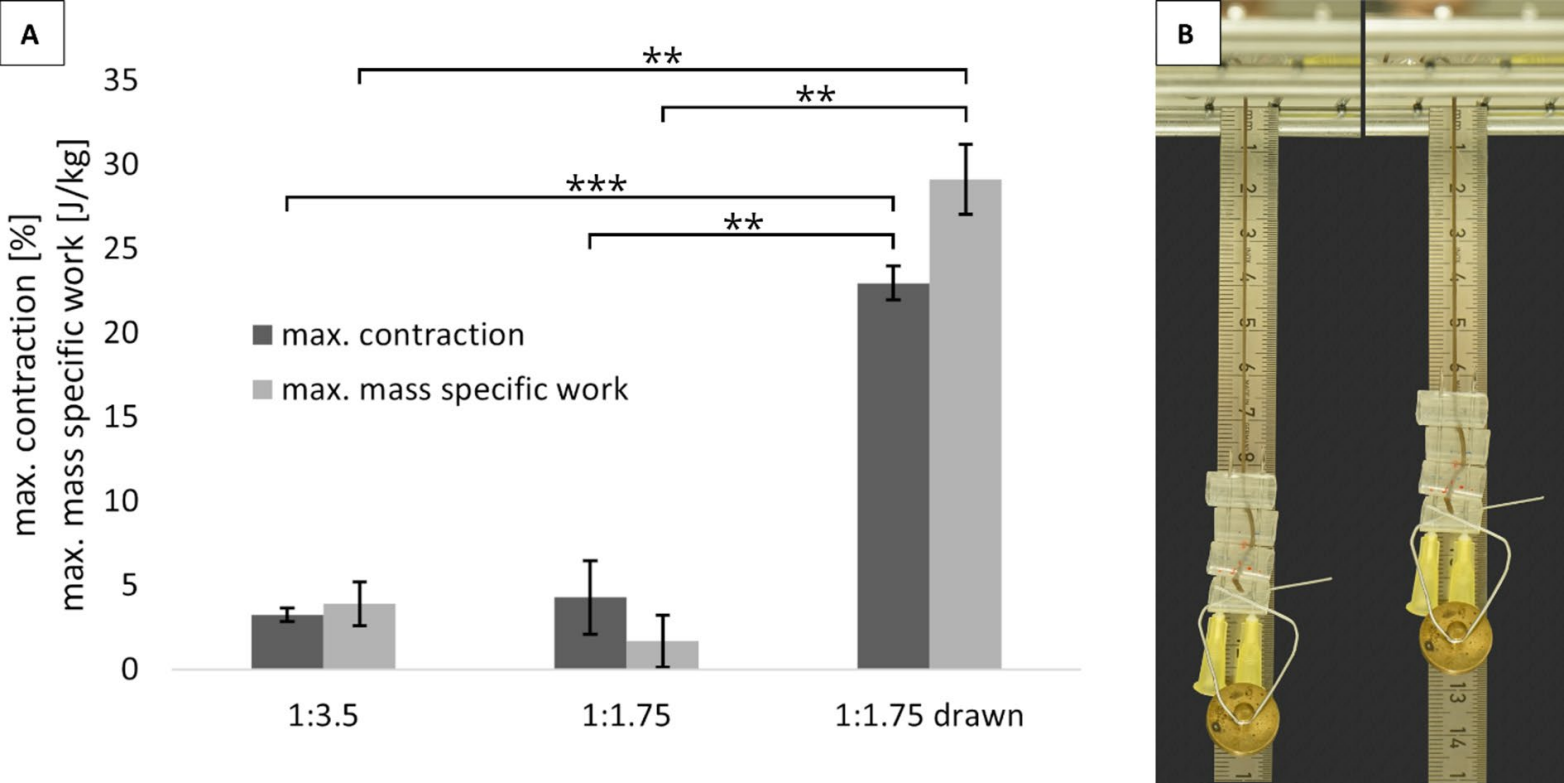
**A** contraction and mass specific work performed of the LCEF with varying molar LC: elastomer ratio and post treatment after activation in a heat chamber, *n* = 5, statistical significance determined by a two-tailed t test with confidence intervals of α = 0.05 [*], 0.01 [**], and 0.001 [***]; **B** experimental setup before (left) and after (right) heating above T_NI_.

Using the experimental setup described in^28^, LCEF 1:3.5 were able to lift 136.32 ± 48.06 times their own weight (0.058 ± 0.013 g) as a result of the nematic-isotropic phase transition without breakage. Thereby, contractions of 3.24 ± 0.38% and mass specific work of 3.899 ± 1.308 J kg^−1^ could be achieved. Higher loads led to failure at the heated state, which occurred due to the softening of the LCEF above T_NI_ (Fig. 2).

The LCEF case group 1:1.75 with halved elastomer content achieved comparable contractions and specific work, but the weight moved was significantly lower. On average, the fibers were able to move only 55.21 ± 31.12 times their own weight (0.038 ± 0.011 g). They achieved contractions averaging at 4.28 ± 2.18%, whereby the contraction capacity increased with increasing additional weight, as with LCEF 1:3.5, showing that mesogen orientation was enhanced by elastic deformation resulting from the attached weight. Specific work averaged at 1.687 ± 1.554 J kg^−1^.

Post-drawing of the LCEF, including a drying process under tension, significantly changed the contraction behavior (Fig. 2). As a result of the post-treatment, the LCEF achieved contractions of 22.96 ± 1.00%, which were at an almost constant level regardless of the additional weight, underlining the high mesogen alignment achieved by post-treatment. Mass-specific work increased with increasing weight up to 29.111 ± 2.083 J kg^−1^, with individual fibers achieving values of up to 31.194 J kg^−1^. The post-drawn LCEF of case group 1:1.75 were able to lift on average 128.66 ± 7.58 times their own weight (0.063 ± 0.026 g). With regard to standard deviation of the determined values, post-drawing strongly homogenized the generated LCEF.

LCE materials based on MBB, 11UB and PMHS are already known in the literature as films and achieve mass-specific work between 0.21 J kg^−1^^39^ and 11.45 J kg^−1^^40^, whereby an average of 3.46 J kg^−1^ was achieved^39–43^. Thus, the LCEF produced in this work without post-treatment have comparable values with respect to the specific working capacity of the LCE films known in the literature. Through post-drawing, however, values could be increased to up to 31.2 J kg^−1^ and thus literature values for MBB-based LCEF were surpassed by a factor of at least 3. However, these MBB-films achieved higher contractions of up to ~ 33%^39–43^, i.e., about 1.5 times more than the post-drawn LCEF produced. Here, especially mesogen orientation play a crucial role for maximizing contraction potential. To estimate LCE network stiffness and thus the ability of mesogen orientation during spinning and post-drawing, swelling experiments were conducted to determine the average number of mesogens between network crosslinks. The results clearly show that the molar mass and corresponding number of PMHS monomer units between crosslinking points in the LCE decrease significantly (*statistical significance determined by a two-tailed t-test with confidence interval α = 0.001*) as the elastomer content increases (24.132 ± 3.449 PMHS monomers and 6.560 ± 0.372 PMHS monomers for 1:1.75 and 1:3.5, respectively); the networks thus become denser and consequently stiffer^30,44,45^. For pos-drawing, no significant difference of network density was determined. In addition to the stiffness of the network and thus its resistance to deformation, the molar ratio has another decisive influence on the actuator properties of the LCE: there must be sufficient space between the network nodes to couple the mesogens as side chains to the elastomeric backbone and rotate them during activation. Thus, the molar ratio between LC and elastomer defines not only the stochastic probability that an LC will couple to the elastomer, but also the network density and thus the strength of the interaction between mesogens and the backbone, which can be influenced by the number of mesogens between network nodes as well as the network stiffness^46,47^. Thus, high elastomer content reduces the interaction of the mesogens with the elastomeric backbone and, consequently, the actuator properties. Further studies need to deeply investigate this phenomenon through detailed characterization of molecular orientation of the mesogens according to LCE composition and during different steps of the spinning process via X-ray diffraction.

For practical application as a muscle substitute, the mass-specific power of the LCEF is particularly relevant, which should be in the range of human muscles (50–284 W kg^−1^^48^ or even exceed it. Therefore, in addition to the specific work generated during activation, contraction time was determined using a static setup utilizing a thermal imaging camera (load-free). Here, contraction times of 0.466 s were determined, correlating to activation frequencies of approx. 2 Hz. Hence, mass-specific powers of the LCEF of 8.28 ± 2.83 W kg^−1^ (1:3.5), 3.62 ± 3.33 W kg^−1^ (1:1.75), and 45.88 ± 23.75 W kg^−1^ (1:1.75 drawn) were calculated. Thus, the generated LCEF with post-drawing can set the values of human muscles and exceed literature values of MBB-based films (mean value 0.26 ± 0.29 W kg^−1^, maximum 0.78 W kg^−1^^39–43^ by a factor of > 60.

### Cytocompatibility

Cytocompatibility is an essential prerequisite for all materials that are to be used on or in the human body. As the LCEF are being developed for potential application as artificial skeletal and cardiac muscles, cell culture studies were necessary to characterize cell behavior after contact with the LCEFs. Furthermore, comparison with RM82-based LCEF from literature was performed to identify differences on biological behavior of the most commonly used.

**Fig. 3:**
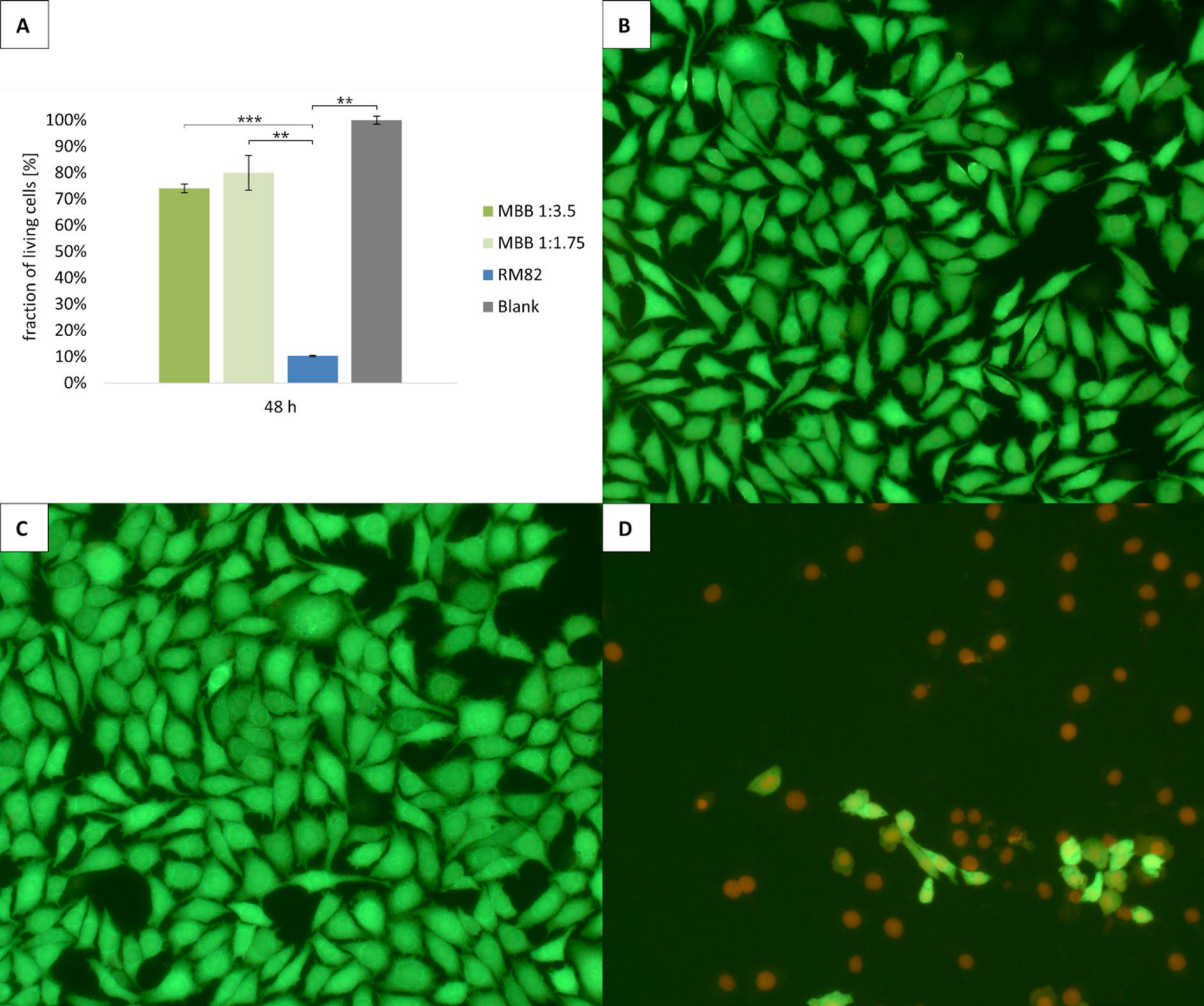
fraction of live cells after cultivating fibroblasts on different LCEF case groups for 48 h determined by XTT (**A**) and live-dead imaging of MBB 1:3.5 (**B**), MBB1:1.75 (**C**), and RM82 (D); *n* = 2 (whereas 3 individual fibers were incubated for each of these 2 extracts); statistical significance determined by a two-tailed t-test with confidence intervals of α = 0.05 [*], 0.01 [**], and 0.001 [***].

LCEF and the generated MBB-based fibers. Here, the material composition was prepared as mentioned by Forman et al.^22^, which is representative for most LCEF presented in literature.

Prior to cell culture experiments, LCEF were incubated in ethanol overnight. Ethanol serves as a swelling agent through which unreacted reagents remaining in the LCE network can diffuse out^49^. Preliminary tests showed that this step was necessary as the LCEF contain unreacted reactants that react cytotoxically. Regardless of their elastomer content, MBB-LCEF extracts exhibited cell viability > 70%, demonstrating cytocompatibility in accordance with DIN EN ISO 10993-5^29^. In contrast, the extracts generated from RM82-based LCEF led to almost no cell survival after 48 h of cultivation. Additionally, live-dead images of the cells after 48 h of cultivation were prepared (Fig. 3B–D), showing nicely spread cells in MBB case groups with rarely any dead cells, whereas cells in the RM82 case group are mostly spherical with destroyed cell walls. Therefore, only MBB-based LCEF are suitable for use in and on the human body and thus for the intended application as a functional muscle substitute.

Since cytocompatibility was tested using extracts of the fiber materials, additionally contact angle measurements against water were conducted to better estimate cellular interaction of the MBB-based LCEF. All case groups demonstrated neither hydrophilic nor hydrophobic behavior (contact angels were 91.92 ± 2.46 °, 83.79 ± 4.55 °, and 95.65 ± 5.95 ° for 1:3.5, 1:1.75, and 1:1.75_drawn, respectively) with no statistically significant differences between case groups. Generally, hydrophilic surfaces are preferred for biomaterials as they foster cell adhesion and reduce e.g., biofouling and cell regulation^50^. Nevertheless, for the intended application as artificial muscle a loose tissue integration is beneficial to avoid restrictions in contractability of the LCEF.

## Conclusions

On the basis of the spinning method presented in^28^ and targeted material developments, LCEF with a human skeletal muscle-like mass specific power and contractability were achieved. Through modification of the molar ratios between the liquid crystal MBB and the elastomeric backbone PMHS as well as the post treatment of the fibers, the generated mass specific work after thermal activation of the nematic-isotropic transition reached values of up to 31.2 J kg^−1^ while activation time was as fast as 0.466 s, resulting in an average mass specific power of 45.88 ± 23.75 W kg^−1^. In comparison, human skeletal muscle achieves values of 7.7–38.57 J kg^−1^ and 50–284 W kg^−1^, respectively^48,51,52^. Most importantly, cell cultivation studies in accordance with DIN EN ISO 10993-5 confirmed the cytocompatibility of the MBB-based LCEF. In contrast, other LCEF materials based on RM82, as commonly used in the literature, were highly cytotoxic. Therefore, the presented MBB-based LCEF show high potential as a biomaterial for use in muscle replacement therapies. Further studies will focus on an in-depth analysis of the generation of mesogen orientation during the spinning process using XRD and enhancing actuation properties, especially in regard to lowering activation temperature and enhancing actuation frequency, thus creating a material suitable for cardiac assistive devices.

## Data Availability

The data that support the findings of this study are available from the corresponding author upon reasonable request.
